# Safety of intravenously applied mistletoe extract – results from a phase I dose escalation study in patients with advanced cancer

**DOI:** 10.1186/s12906-017-1971-1

**Published:** 2017-09-18

**Authors:** Roman Huber, Dietrich Schlodder, Carola Effertz, Sabine Rieger, Wilfried Tröger

**Affiliations:** 1 Center for Complementary Medicine, University Medical Center Freiburg, Faculty of Medicine, University of Freiburg, Breisacher Str. 115B, 79106 Freiburg im Breisgau, Germany; 2 Helixor Heilmittel GmbH, Rosenfeld, Germany; 3 Clinical Research Dr. Tröger, Freiburg, Germany

**Keywords:** *Viscum album*, Intravenous, Cancer, Clinical trial, MTD, Side effects

## Abstract

**Background:**

Mistletoe extracts have anti-tumor properties and are approved for subcutaneous use in cancer patients. Data on Intravenous application are limited.

**Methods:**

An aqueous extract from pine-mistletoe was used to investigate maximum tolerable dose (MTD) and safety of intravenous application. It was infused once weekly for 3 weeks in patients with advanced cancer. Any type of cancer was included; relevant exclusion criteria were concurrent chemo- or radiation therapy. The classical phase I 3 + 3 dose escalation scheme was followed. Predefined dose groups were 200, 400, 700, 1200 and 2000 mg. Maximum planned dose was 2000 mg. With the MTD three more patients should be treated for 9 weeks in order to evaluate intermediate term tolerability. Weekly during the treatment and 1 week later tolerability, clinical status, safety laboratory parameters and adverse events were documented.

**Results:**

Twenty-one patients (3 in the dose groups 200, 400, 700 and 1200 mg, respectively, 9 in the dose group 2000 mg) were included. MTD was not reached. Because one dose-limiting toxicity (DLT), an allergic reaction, occurred during infusion of 2000 mg, three more patients had to be included in this dose group and tolerated it, as well as the three patients who received 2000 mg for 9 weeks. Occasionally in the dose group 2000 mg mild to moderate fever occurred.

**Conclusion:**

Weekly infusions of 2000 mg of the pine-mistletoe extract were tolerated and can be used in further studies but had a risk for allergic reactions and fever. German Clinical Trials Register (Trial registration number DRKS00005028).

## Background

*Viscum album* is a species of mistletoe that occurs predominantly in central Europe, growing as a semi-parasitic plant on various host trees. Extracts from *Viscum album* (VaE) have been used since decades in complementary cancer therapy, especially within the concept of anthroposophic medicine. They contain various substances with biologic activity (mistletoe lectins (ML), viscotoxins, polysaccharides, flavonoids, phenylpropanoids, triterpenoids and others) of which ML have been found most interesting. Mistletoe extracts and ML have shown to possess distinct cytotoxic effects on a panel of 38 cancer cell lines with low IC_50_ values (0.026 μg/ml) for ML [1]. In addition to cytotoxicity, VaE and ML have shown immunomodulatory properties, which are mediated by an activation of dendritic cells [2]. After subcutaneous application in humans, VaE increased the number of T-cells, eosinophils and neutrophil granulocytes in comparison to placebo [3, 4] and there is some evidence from randomized, placebo-controlled clinical trials that quality of life of cancer patients during chemo- or radiation therapy is improved [5]. Therefore, subcutaneously applied standardized VaE are approved in Germany and other countries for supportive therapy in cancer patients irrespective of cancer type [6, 7]. Because of local reactions at the site of injection, which are related to the amount of ML in VaE [3, 4], tolerability of doses with systemic anticancer activity is limited by this route of application; it causes relevant pain, nausea and fever [8]. Absorption of ML from the gastrointestinal tract has been shown in an in vitro model with M-cells [9], but overall absorption of ML after oral application in humans seems to be poor, because they interact with carbohydrate residues on epithelial cells [10, 11] and no studies in humans have been performed with orally applied VaE. Therefore, the intravenous route is interesting to apply cytotoxic concentrations of ML, but so far data for this route of application are very limited. In an observational study off label VaE infusions have been regarded as safe but some patients reported fever or pruritus [12]. Single controlled studies reported prevention of immunosuppression related to chemotherapy [13] or operative stress [14, 15]. In a small RCT (*n* = 64) patients with colorectal cancer had prolonged survival with postoperative chemotherapy and i.v. VaE compared to chemotherapy alone [16]. The maximum tolerable dose of i.v VaE is unknown. Because of the promising in vitro cytotoxicity of ML and first reports about clinical effects and good safety of i.v VaE in cancer patients, we systematically wanted to investigate dose related safety and maximum tolerable dose of a VaE and performed a prospective phase I clinical trial.

## Methods

### Study medication

The mistletoe product Helixor® P (Helixor Heilmittel GmbH, Rosenfeld; Germany) was used for the study. Helixor® P is registered in Germany since 1982 as an anthroposophic medicinal product for subcutaneous injection [17]. It is derived from mistletoes growing on pine trees. One ampoule of 2 ml solution for injection contains the extract of 100 mg of fresh mistletoe herb (drug to extract ratio is 1:20). Extracting agent is water for injection, sodium chloride (99.91:0.09). This 100 mg extract contains about 1000 ng/ml ML-III. For intravenous infusion the respective dose was diluted with 250 ml physiologic saline solution and infused in three hours’ time. The rationale for using Helixor® P was, that some beneficial clinical experiences have been reported with this extract [18, 19] and that it has a high content of ML.

### Study design

The prospective, dose-escalating, phase I GCP study without control group was performed at the Center for Complementary Medicine, University Medical Center Freiburg, Germany. The classical phase I 3 + 3 dose escalation scheme was followed. Predefined dose groups were 200, 400, 700, 1200 and 2000 mg. If three patients tolerated three infusions of the first dose (200 mg) in weekly intervals, the next three patients received three infusions of the next dose (400 mg) and so forth. Maximum planned dose was 2000 mg. If the maximum dose was tolerable three more patients should be treated weekly for 9 weeks in order to evaluate intermediate term safety. The choice of the starting dosage (2 ampoules of Helixor® P 100 mg/2 ml) is based on above mentioned clinical experiences, not showing any side effects below this dosage. The choice of the highest dosage (20 ampoules of Helixor® P 100 mg/2 ml) is derived from the demand of limiting the endotoxin level in solutions for intravenous infusions to 5 IU per kg body weight per hour. The internal specification for endotoxins is ≤24.0 IU/ml Helixor® P. Infusion of 2000 mg Helixor® P in a patient of 60 kg body weight accordingly should take 3.2 h. Based on experiences with i.v. mistletoe treatment, an increased rate of febrile reactions and allergic or pseudoallergic reactions has been expected [12].

### Objectives

Primary objective was to determine the maximum tolerated dose (MTD) of i.v. Helixor® P. The MTD is based on the incidence of dose-limiting toxicities (DLTs). Secondary objectives were to investigate safety and tolerability of the different dosages and the clinical course of the patients.

### Study population

Female and male patients ≥18 years of age with a histological or cytological confirmed diagnosis of an advanced malignant disease in an interval without antineoplastic therapies with an ECOG: performance status 0–2 and sufficient bone marrow function, defined as leucocytes ≥3000/mm^3^, neutrophils ≥1500/μl, and thrombocytes ≥100,000/mm^3^ in the peripheral blood were included. Exclusion criteria were severe concomitant diseases (e.g. cardiovascular, respiratory, autoimmune disease needing immunosuppressants, severe allergic illness), fever, hyperthyreoidism, known hypersensitivity to mistletoe products, preceding therapy with mistletoe products, creatinine level > 1.5 mg/dl, bilirubine level > 3 x upper limit of normal, aminotransferase levels >3 x upper limit of normal, planned or current therapy with surgery, radiotherapy or chemotherapy, known abuse of medicaments, alcohol or illegal drugs, pregnancy or breast feeding, participation in another clinical study and dependence in relation to the sponsor’s or investigator’s institutions. Informed consent was obtained of all patients before inclusion in the study.

### Quality assurance

The study was conducted in accordance with the Declaration of Helsinki, with the International Conference on Harmonization (ICH) Good Clinical Practice Guidelines and the German drug law. It was approved by the German health authorities (BfArM), and registered in the European Union (EudraCTno, 2012–004189-16) and the German Clinical Trials Register Freiburg (Trial registration number DRKS00005028, date of registration May 8th, 2013). The positive vote of the responsible ethical committee (University of Freiburg, Germany) was obtained before onset of the study (EK 93/13). All study procedures followed the clinical study protocol and the Standard Operating Procedures (SOPs) of the sponsor, the data management, and the CRO which are based on the current regulatory and ethical requirements. In addition, a study-specific monitoring guideline, a data management plan, and a statistical analysis plan had been prepared. The conduct of the study was supervised by specifically trained clinical monitors.

The study personnel of the study site was instructed by the clinical monitor in the clinical study protocol, the study procedures, GCP, and administrative and regulatory procedures (e.g. reporting of serious adverse events) during a study initiation visit. After study initiation, the clinical monitor performed 15 monitoring visits.

### Adverse events (AEs), dose limiting toxicity (DLT) and maximum tolerable dose (MTD)

Any untoward medical occurrence, irrespective of the relationship to the study medication or study procedure, were recorded as AE in the CRF, assessed on its grade of severity, causality to the study medication, clinical consequences and outcome and classified as serious (SAE), suspected unexpected serious (SUSAR), if applicable. Dose limiting toxicity (DLT) was defined as a specific AE ≥ grade 2. Grading of specific AEs expected during MI were

- Grade 1: Transient rash, drug fever ≤39,8 °C for less than 24 h
- Grade 2: Urticaria, and/or asymptomatic bronchospasm, drug fever >39,8 °C or drug fever >39.5 °C continuing more than 24 h
- Grade 3: Symptomatic bronchospasm, requiring parenteral medication(s), with or without urticaria; allergy-related edema/angioedema
- Grade 4: Anaphylaxis

Also an unexpected increase of aminotransferase levels to >3× baseline levels or any CTCAE ≥ grade III, if a causal relationship cannot be ruled out and happened between the 1st and one week after the last application was assessed as DLT. The MTD is defined as the highest dose applied within this study that provokes a DLT at a maximum probability, defined prior to study opening. In analogy to most MTD-finding studies and corresponding to the pretended toxicity threshold of the 3 + 3 design, a DLT maximum probability of 33% was chosen for this trial [20].

### Safety and tolerability assessments

Each patient received a complete physical examination at screening visit and at study exit. Weekly during the study, the patient was briefly examined (vital signs, weight, examination of areas of pre-existing problems, temperature). An electronic thermometer and a patient diary were given to the patients to measure body temperature at about 8:00 am 2:00 pm and 8:00 pm at home daily.

Weekly white and red blood cell count, bilirubin, creatinine, aspartate and alanine aminotransferase (AST, ALT), gamma glutamyl transferase (γGT), alkaline phosphatase (AP), albumin, chloride, sodium, potassium, calcium, uric acid, and prothrombin time were checked.

Tolerability was rated by the patient in the patient diary at day 1 and day 2 after the infusion on a 5-point scale: very good, good, moderate, bad, and very bad.

### Sample size, statistics and data management

All data from the CRF were recorded into the ACCESS® database by input masks reflecting content and layout of the CRF forms. Independent double data entry with third party reconciliation was used; data were validated according to a validation plan. Medical coding used MedDRA for concurrent diseases and adverse events, and WHO-DD for therapies and concomitant medications. Sample size of this dose finding phase I study was planned according to three scenarios which showed that between a minimum of 6 and a maximum of 33 patients had to be included.

Three analysis populations (full analysis set (FAS), safety analysis set (SAS) of patients, who at least received one MI, and per protocol set (PPS) of subjects without major protocol violations) were defined and analysed separately. Missing data were not imputed. Primary analysis was descriptive, safety and tolerability were analysed with explorative statistics by the statistical program IBM SPSS Statistics Professional.

## Results

From 93 patients who were screened for the study, 72 had to be excluded, mostly because chemotherapy was planned or because they had been pre-treated with mistletoe preparations. 21 patients with advanced or metastasized cancer were included. Table 1 shows the types of cancer and characteristics of the patients at baseline. Most patients were pre-treated with surgery, chemotherapy and radiotherapy. All patients were Caucasians and had no chemo- or radiation therapy during the study period. 3 patients each were treated with 200, 400, 700, 1200 and 2000 mg Helixor® P for three weeks, respectively (Table 2). There was no drop out. The last patient in the dose group 2000 mg developed a dose limiting toxicity (DLT, allergic reaction with generalized urticaria requiring intravenous antihistamines) during the infusion. According to the protocol, three more patients were treated with this dose for three weeks and tolerated it without DLT. Because no DLT was reached, three more patients were treated with the maximum dose 2000 mg for 9 weeks to evaluate intermediate term safety.

**Table 1:** Patient characteristics

Characteristics	
Age in years (median and range)	64 (42–74)
Gender (number of male and female patients)	17 / 4
ECOG-performance status (number of patients)	
0	5
1	12
2	4
Primary tumour (number of patients)	
Prostate cancer	4
Colorectal cancer	3
Renal cell cancer	2
Hepatocellular cancer	2
Sarcoma	2
Glioblastoma	1
Lung cancer	1
Stomach cancer	1
Pancreatic cancer	1
Gall bladder cancer	1
Tonsil cancer	1
Thyroid (C-cell) cancer	1
Thrombocythemia	1
Previous treatment (number of patients)	
Surgery	15
Chemotherapy	14
Radiotherapy	9
Immunotherapy	4
Time since primary cancer diagnosis (years)	6.7 ± 8.8
Body mass index (kg/m2)	24.2 ± 5.6
Blood pressure (mmHg)	126 ± 18 / 82 ± 11
Puls rate (Beats/min)	72 ± 13
Rectal temperature (°C)	36.6 ± 0.5

**Table 2:** Treatment with Helixor® P and dose limiting toxicity (DLT)

Dose in mg	Number of patients	Treatment duration (weeks)	DLT
200	3	3	No
400	3	3	No
700	3	3	No
1200	3	3	No
2000	3	3	Yes (patient 15)
2000	3	3	No
2000	3	9	No

### Safety and tolerability

All patients were evaluated for safety at each visit. In general, Helixor® P was well tolerated. Tolerability of 2000 mg was not different from 400 mg (Table 3) and none of the infusions was rated as bad or very bad tolerable.

**Table 3:** Patients’ rated tolerability of infusion

Rating	200 mg	400 mg	700 mg	1200 mg^a^	2000 mg^a^
Number of infusions rated	9	9	9	9	27
Very good	100%	33%	0	44%	33%
Good	0	22%	56%	33%	33%
Moderate	0	44%	44%	0	26%
Bad	0	0	0	0	0
Very bad	0	0	0	0	0

^a^2 missing values

Six serious adverse events (SAE) occurred during the study. All were related to a progress of the disease or other reasons (e.g. hospital admission because of dyspnoe due to pulmonary metastases, change of a bile duct stent due to cholestasis, hospital admission because of dizziness after sudden increase of opiate medication) and not to the study medication. None of the SAE resulted in discontinuation of the study medication. No suspected unexpected serious adverse event (SUSAR) occurred.

One hundred fifty-five adverse events (AE) were documented and classified during the study. No clear dose dependency of the number of AE could be found (Table 4).

**Table 4:** Number of adverse events (AE) in relation to dose administered (*n* = 155)

Dose (mg)	200	400	700	1200	2000^a^	2000^b^	2000^c^
Clinical AE (n)	2	0	6	3	16	7	1
Laboratory AE (n)	16	16	16	20	14	12	25

^a^ *n* = 3 before DLT; ^b^ *n* = 3 after DLT; ^c^ *n* = 3 for 9 weeks

**Fig. 1:**
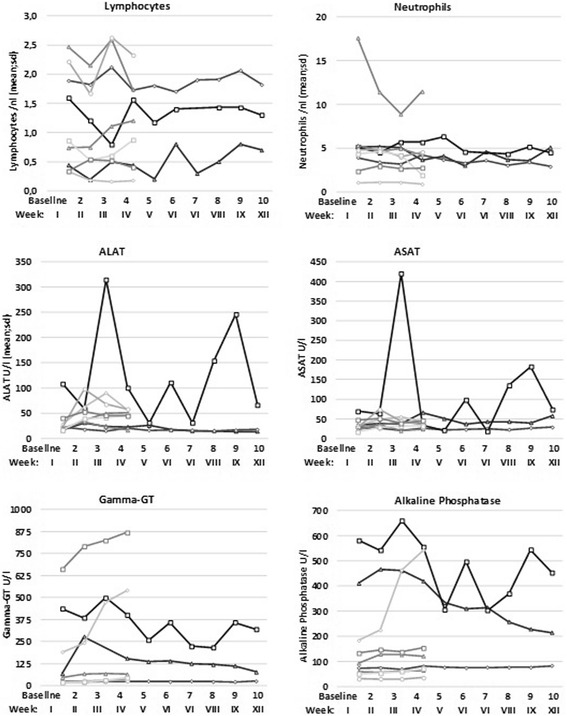
Course of selected laboratory parameters (neutrophils, lymphocytes, alanine aminotransferase, aspartate aminotransferase, alkaline phosphatase, gamma-glutamyltransferase) of all nine patients (6 treated for 3 weeks, 3 treated for 9 weeks) in the 2.000 mg dose-group. Arabic figures indicate the number of visit

Twenty of the 155 AE have been classified as at least possibly related to the study medication, all of them occurred in the dose group 2000 mg. These were apart from the allergic reaction in one patient mentioned above: grade 1 fever in 4 patients, weakness the day after infusion (*n* = 3), eosinophilia (up to 19% in 2 patients), and slight temporary elevation of alanine aminotransferase (ALT) in two patients. Mean levels of ALT, aspartate aminotransferase (AST) and alkalic phosphatase (AP) and neutrophil or lymphocyte counts (Fig. 1) were not changed. In none of the patients with AE, except the one with allergic reaction, the study medication was changed.

### Clinical observations

The DLT occurred in a 62-year-old male patient with prostate cancer recurrence. He was obese (Body mass index 31.5), had no history of allergic diseases and had normal ALT levels at baseline. In the night after the first infusion (2000 mg) he developed fever and ALT levels increased from 22 to 98 U/l one week later without concomitant change of gamma glutamyl-transferase and alkaline phosphatase. The increase of ALT was regarded as possibly related to the study medication. During the end of the second infusion he developed mild rash and itching at the abdomen, during the third infusion he within 15 min developed generalized urticaria which required injection of 2 mg clemastinfumarate. After 1 h observation and continuous improving he was sent home. In the nights following infusion 2 and 3 he had subfebrile temperature. ALT levels one week after infusion 2 and 3 decreased to 68 and 58 U/l, respectively.

**Fig. 2:**
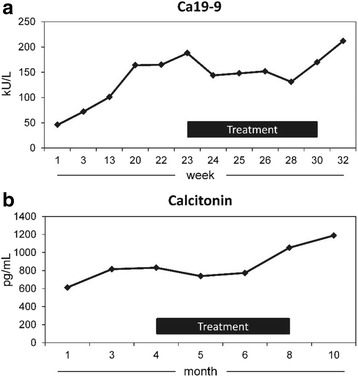
Tumor markers of a patient with pancreatic cancer (**a**) and a patient with metastasized C-cell carcinoma (**b**) before, during and after treatment with 700 mg (**a**) and 2000 mg (**b**) Helixor® P. After 3 weeks in the study, treatment was continued off label until tumor markers increased again

Two patients had unexpected temporary improvement of tumor markers during and after the end of the study. A 50-year-old patient with pancreatic cancer and lung metastases had a reduction of Ca19–9 for 5 weeks during treatment with 700 mg Helixor P (Fig. 2a). One 72-year-old patient with metastasized C-cell carcinoma had a stable course of Calcitonin for two months and reported about marked improvement of his pre-existing fatigue during treatment with 2000 mg (Fig. 2b). Both patients were treated off label with the respective dose (700; 2000 mg) until the tumor marker increased again. Both patients had not received any other anti-tumor treatment the weeks before and during this period.

One 56-year-old patient with peritoneal carcinomatosis and abdominal wall metastases from colorectal cancer has had computed tomography (CT) documented tumor progression with Panitumumab before inclusion into the study. CT controls 3 and 6 months during study and off label treatment with 2000 mg Helixor® P weekly showed stable disease. After 9 months the patient had slow radiological progression and after further slow progression 3 months later (1 year after start of treatment), treatment was changed to intra-tumoral mistletoe injections. The patient is still alive 2.5 years after she entered the study but has slow continuous progression. She regularly visited oncologic surgeons and oncologists from the Center for Gastrointestinal Tumors of University Medical Center Freiburg but no other anti-tumor treatment than mistletoe preparations have been given since she entered the study because the tumor was not operable and chemotherapy was refused from her.

## Discussion

This is the first phase I study investigating the MTD of a mistletoe preparation (MP) and the first investigating the intravenous route of application. A DLT was not reached. Infusion of the pre-defined maximum dose of 2000 mg Helixor® P, which contains among others 40,000 ng natural ML III, has a certain risk of allergic reactions and provoking short term (<12 h) elevated body temperature, but does not seem to have bone marrow or other organ toxicity.

In a pharmacokinetic study a single subcutaneous injection of a mistletoe preparation containing 20,000 ng natural ML I caused fever and fever related symptoms in all probands [8]; up to 10 ng/kg of recombinant ML had similar side effects [21]. Repetitive subcutaneous injections of ML containing mistletoe products are furthermore known to cause blood eosinophilia [3, 4] and allergic reactions are known as possible side effects (Helixor® investigator’s brochure). It seems, therefore, that the spectrum of side effects form ML containing MP is the same after subcutaneous and intravenous route of application. Administration of higher doses than 2000 mg Helixor® would have offended against the rules of the European Pharmacopoeia, Chapter 2.6.14. “Bacterial Endotoxins”, due to an endotoxin level exceeding the regulations. Although the endotoxin-analysis is not reliable, due to a cross-reaction with mistletoe-lectins in the limulus-test [22], no higher doses have been investigated in the study.

In the one patient who had an ALT increase, which was regarded possibly related to the study medication, a direct hepatotoxicity of the IMP is unlikely, because a further increase instead of decrease during continued treatment would have had to be expected. Fever can increase ALT levels [23] and the abnormality can therefore best be explained by fever which was induced by the IMP. None of the patients treated with Helixor® P 2000 mg for 9 weeks showed abnormalities in liver enzyme in comparison to baseline. Also in an observational study in which various concentrations of Helixor® P where used intravenously, no changes of liver enzymes have been reported [12].

Interestingly, three patients had temporary stable courses of previously deteriorated tumor markers or imaging. Whether this was related to an anticancer effect of the study medication can due to the uncontrolled design of our study not be proven. Because no other anticancer therapies have been applied during this time and they had had progression of their disease in the months before entering the study a causal relationship is at least possible. Remission was not assessable as outcome parameter in this phase I study lasting 4–9 weeks for the individual patient only.

Taking together the good safety profile and possible clinical anticancer effects the parenteral application of the IMP deserves future investigations. There are no indications of any new potential side effects related to intravenous infusions of the IMP when compared to the subcutaneous route of application. The data pave the way for a standardized, randomized, placebo-controlled, phase-III-trial testing efficacy of misteltoe, which is urgently needed and essential.

## Conclusions

Weekly infusion of the mistletoe product Helixor® P was tolerated up to a starting dose of 2000 mg. In this dose group an increased risk for side effects (allergic reaction, fever) has to be expected. Single patients had unexpected reductions of tumor markers or stable disease.

## Abbreviations

AE: Adverse event
CRF: Case report form
CRO: Contract Research Organisation
CT: Computed tomography
CTCAE: Common toxicity criteria for adverse events
DLT: Dose-limiting toxicity
DMC: Data Monitoring Committee
ECOG (−performance status): Eastern Cooperative Oncology Group
FAS: Full analysis set
IMP: Investigational medicinal product
ML: Mistletoe lectins
MP: Mistletoe preparation
MTD: Maximum tolerable dose
PPS: Per protocol set
RCT: Randomised clinical trial
SAE: Serious adverse event
SAS: Safety analysis set
SOP: Standard operating procedure
SUSAR: Suspected unexpected serious adverse reaction
VaE: *Viscum album* extract

## References

[1] Kelter G, Schierholz JM, Fischer IU, Fiebig HH (2007). Cytotoxic activity and absence of tumor growth stimulation of standardized mistletoe extracts in human tumor models in vitro. Anticancer Res.

[2] Elluru SR, Duong van Huyen JP, Delignat S, Kazatchkine MD, Friboulet A, Kaveri SV, Bayry J (2008). Induction of maturation and activation of human dendritic cells: a mechanism underlying the beneficial effect of Viscum album as complimentary therapy in cancer. BMC Cancer.

[3] Huber R, Rostock M, Goedl R, Lüdtke R, Urech K, Buck S, Klein R (2005). Mistletoe treatment induces GM-CSF- and IL-5 production by PBMC and increases blood granulocyte- and eosinophil counts: a placebo controlled randomized study in healthy subjects. Eur J Med Res.

[4] Huber R, Ludtke H, Wieber J, Beckmann C. Safety and effects of two mistletoe preparations on production of Interleukin-6 and other immune parameters - a placebo controlled clinical trial in healthy subjects. BMC Complement Altern Med. 2011, 11(1):116. 10.1186/1472-6882-11-116 PMC3257204 22114899

[5] Horneber MA, Bueschel G, Huber R, Linde K, Rostock M. Mistletoe therapy in oncology. Cochrane Database Syst Rev. 2 edn. 2008:CD003297. 10.1002/14651858.CD003297.pub2 PMC7144832 18425885

[6] Summary of Product Characteristics of the mistletoe preparation Helixor. https://www.gelbe-liste.de/produkte/helixor-p_353598/fachinformation

[7] Drug guidelines of the German Federal United Commission https://www.g-ba.de/downloads/83-691-323/AM-RL-I-OTC-2013-06-05.pdf

[8] Huber R, Eisenbraun J, Miletzki B, Adler M, Scheer R, Klein R (2010). Pharmacokinetics of natural mistletoe lectins after subcutaneous injection. Eur J Clin Pharmacol.

[9] Lyu SY, Park WB (2010). Mistletoe lectin transport by M-cells in follicle-associated epithelium (FAE) and IL-12 secretion in dendritic cells situated below FAE in vitro. Arch Pharm Res.

[10] Büssing, A. Biological and pharmacological properties of *Viscum album* L., In Mistletoe, The Genus Viscum. Büssing, A. (Ed.). Harwood Academic Publishers, Amsterdam, The Netherlands, pp. 123–182, (2000).

[11] Ziska P, Gelbin M, Franz H, Van Driesche E, Franz H, Beeckmans S, Pfüller U, Kallikorm A, Bog-Hansen TC (1993). Interaction of mistletoe lectins ML I, ML II and ML III with carbohydrates. Lectins: biology, biochemistry, clinical biochemistry.

[12] Steele ML, Axtner J, Happe A, Kröz M, Matthes H, Schad F. Safety of Intravenous Application of Mistletoe (*Viscum album* L.) Preparations in Oncology: An Observational Study. Evid Based Complement Alternat Med. 2014:1–10. doi:10.1155/2014/236310. 10.1155/2014/236310 PMC4052504 24955100

[13] Büssing A, Brückner U, Enser-Weis U, Schnelle M, Schumann A, Schietzel M (2008). Modulation of chemotherapy-associated immunosuppression by intravenous application of *Viscum album* L. Extract (Iscador): A randomised phase II study. Eur J Integr Med.

[14] Schink M, Tröger W, Dabidian A, Goyert A, Scheuerecker H, Meyer J (2007). Mistletoe extract reduces the surgical suppression of natural killer cell activity in cancer patients. A randomized phase III trial. Forsch Komplementmed.

[15] Büssing A, Bischof M, Hatzmann W, Bartzsch F, Soto-Vera D, Fronk EM (2004). Beeinflussung der Granulozytenfunktion durch einmalige perioperative Mistelextrakt-Infusion. Dtsch Zschr Onkol.

[16] Cazacu M, Oniu T, Lungoci C, Mihailov A, Cipak A, Klinger R (2003). The influence of Isorel on the advanced colorectal cancer. Cancer Biother Radiopharm.

[17] Helixor Heilmittel GmbH. Fachinformation Helixor A/−M/−P Deutschland. In*.*; 2014.

[18] Kalden M (1994). Klinische Erfahrungen mit *Viscum album* bei fortgeschrittenen Tumoren. Erfahrungsheilkunde.

[19] Böcher E, Stumpf C, Büssing A, Schietzel M (1996). Prospektive Bewertung der Toxizität hochdosierter *Viscum album* L.-Infusionen bei Patienten mit progredienten Malignomen. Z Onkol.

[20] Storer BE (1989). Design and analysis of phase I clinical trials. Biometrics.

[21] Bergmann L, Aamdal S, Marreaud S, Lacombe D, Herold M, Yamaguchi T (2008). Phase I trial of r viscumin (INN: aviscumine) given subcutaneously in patients with advanced cancer: a study of the European Organisation for Research and Treatment of Cancer (EORTC protocol number 13001). Eur J Cancer.

[22] Scheer R (1993). Beeinflussung des Limulus-Amöbozyten-Lysat-Tests durch Mistel-Lektine. Arzneim-Forsch/Drug Res.

[23] Kang KS (2013). Abnormality on liver function test. Pediatr Gastroenterol Hepatol Nutr.

